# Age-related vulnerability to sleep deprivation is task dependent and influenced by large inter-individual differences in younger adults

**DOI:** 10.1093/sleep/zsaf144

**Published:** 2025-05-30

**Authors:** Elly Francis-Pester, Jessica E Manousakis, Anna W T Cai, Jinny Collet, Clare Anderson

**Affiliations:** School of Psychological Sciences, Monash University, Clayton, Victoria, Australia; School of Psychological Sciences, Monash University, Clayton, Victoria, Australia; School of Psychological Sciences, Monash University, Clayton, Victoria, Australia; School of Psychological Sciences, Monash University, Clayton, Victoria, Australia; School of Psychological Sciences, Monash University, Clayton, Victoria, Australia; Centre for Human Brain Health, School of Psychology, University of Birmingham, Edgbaston, Birmingham, United Kingdom

**Keywords:** aging, sleep deprivation, attention, sleepiness, performance, task dependency

## Abstract

**Study Objectives:**

Healthy older adults appear more resilient to sleep loss relative to younger adults, particularly with respect to the likelihood of falling asleep. We examined task-dependent differences in age-related vulnerability to sleep deprivation focusing on outcomes reflecting sleep-initiation (microsleep, slow eye movements [SEMs], electroencephalography [EEG] delta power, and long lapses > 3 s) versus other non-sleep-initiation aspects of impairment (adjusted mean RT/lapses, reflexive attention, and inhibitory control).

**Methods:**

Seventeen younger (*M* = 24.5 ± 3.2 years [range 21–33 years], 10 males) and 17 older (*M* = 57.3 ± 5.2 years [range 50–65 years], 9 males) healthy adults underwent 26 h of sleep deprivation. Test batteries (psychomotor vigilance test [PVT], Karolinska drowsiness test, and ocular motor paradigms) with simultaneous EEG were administered at regular intervals throughout.

**Results:**

During sleep deprivation, younger adults had significantly more sleep-initiation events relative to older adults (*p* < .031) including EEG microsleep (average 4.4 vs. 1.1), SEMs (10.7 vs. 4.9), and relative delta power (38.1 vs. 24.2%). For non-sleep-initiation outcomes, interaction effects were not observed. Both younger and older groups had slower reflexive attention (4.2 and 3.1 ms, respectively) and poorer inhibitory control (8.6% and 9% more errors) during sleep deprivation relative to when well-rested (*p* < .001), with older adults being more impaired than younger adults overall (*p* < .001). Large inter-individual differences in sleep-initiation events were observed for younger adults. Preliminary results suggest women exhibited age-related differences in all sleep-initiation events (Hedges’ *g* = 0.73 to 1.64), while men did not (*g* = 0.12 to 0.34).

**Conclusions:**

Younger adults are more likely to fall asleep during sleep deprivation, particularly women, while older adults may be more likely to exhibit attentional control difficulties.

## Graphical Abstract

**Graphical Abstract:**
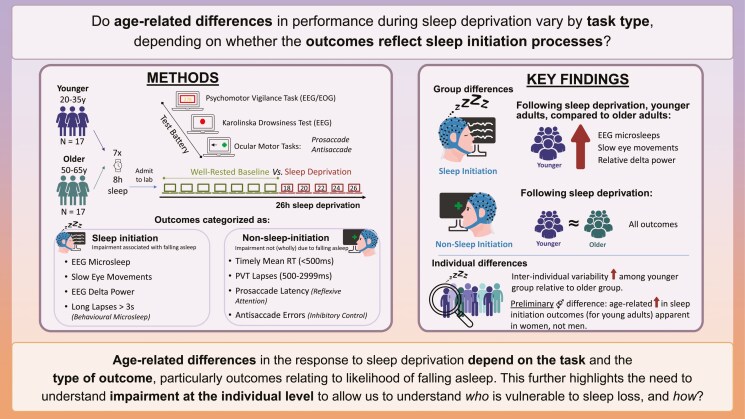

**Statement of Significance**

In industrialized societies, a significant proportion of the population is engaged in shift work with a growing number of older adults. Understanding how *all* adults of working age may be impacted by a night without sleep is critical to keeping the workforce safe and productive. We show both younger and older adults are impaired by sleep deprivation on performance-based outcomes. Younger adults however are more likely to fall asleep, particularly younger women. Future work should systematically examine individual differences in the response to sleep loss across a variety of tasks/outcomes for all ages and other forms of sleep loss. Identifying who is most vulnerable to sleep loss, and importantly how, would provide important information for future mitigation strategies to address the global burden of sleep loss in society.

## Introduction

One night of sleep deprivation has a widespread impact on human performance. This includes impaired alertness and attention, and reduced executive functions such as cognitive flexibility, inhibitory control, and working memory [1–4]. Failure to perform across any of these domains can have significant adverse effects on health, safety, and performance [5], particularly in safety-critical occupational settings, such as transportation [6], healthcare [7, 8], and mining operations [9]. These environments typically involve round-the-clock operations requiring individuals to work throughout the night-time hours resulting in sustained periods of sleep deprivation and subsequent sleepiness [5]. While the cognitive consequences of sleep deprivation in younger adults (e.g. < 30 years) are well understood, particularly concerning sustained attention, much less is known about how older individuals respond to sleep deprivation, especially at the individual level. As the aging population continues to grow, there is a rising trend of older adults remaining in the workforce [10], with many engaging in shift work as frequently as younger adults [11]. Therefore, it is crucial to understand the cognitive consequences of sleep deprivation across these different age groups to ensure healthy, safe, and productive workplaces, particularly in environments that involve overnight work.

Previous studies suggest that healthy older adults are less susceptible to the consequences of sleep deprivation when compared to younger adults. For example, following one night without sleep, younger adults exhibit greater impairment in sustained attention, as indicated by slower response times, more lapses of attention, and more variability in psychomotor vigilance task (PVT) response times, when compared to older adults [12–15]. This impairment is corroborated by physiological markers of sleepiness, whereby during a night without sleep younger adults exhibit more slow eye movements (SEMs) [15] and microsleeps [16], relative to older adults. Taken together, these findings suggest that older adults may tolerate sleep deprivation better than younger adults, particularly when assessing the capacity to remain awake and alert. From a neurobiological perspective, one might expect that older adults would be less likely to fall asleep during periods of sleep deprivation due to neuronal loss in the sleep-initiating ventrolateral preoptic nucleus [17]. However, other neurobiological changes that occur with age suggest that older adults may preferentially exhibit other types of performance impairment during sleep deprivation that are not wholly explained by the tendency to fall asleep. For instance, as the prefrontal cortex appears most vulnerable to both aging [18–20] and sleep deprivation [21–23], one might additionally hypothesize that older adults would show enhanced vulnerability on tasks designed to assess the executive components of attention, such as inhibitory control (e.g. to ignore one stimulus and instead attend to another). Taking this into consideration, capturing attentional impairments due to sleepiness/sleep-initiation versus more performative/executive components of attention may shed some further insight into age-related changes following sleep deprivation. However, although task-dependent effects of sleep deprivation are well described in in younger adults [24–27], there remains a paucity of data examining task-dependent effects of sleep deprivation in older adults. To address this, our study examined age-related differences in response to (total) sleep deprivation across a range of alertness and attention-based tasks typically employed in sleep research, but with a focus on outcomes that reflect sleep initiation (i.e. the process of falling asleep such as eye closure or microsleep) and non-sleep initiation (i.e. performance not wholly explained by sleep initiation such as reflexive attention or inhibitory control). Due to the well-established individual differences in response to sleep deprivation [25, 28, 29], we will examine age-related differences across tasks at both the group (averages for sleep loss and age) and individual (person-by-person) level.

## Methods

### Participants

Thirty-nine participants recruited from the community were enrolled in the study (*n* = 20 younger; *n* = 19 older). Of these, five did not complete the study due to non-compliance with the structured sleep schedule (*n* = 2 younger) or withdrawal during the in-lab sleep deprivation protocol (*n* = 1 younger, *n* = 2 older). Thirty-four participants completed the study: 17 healthy young adults aged 21 to 33 (24.5 ± 3.2 years; 10 males) and 17 healthy older adults aged 50 to 65 (57.3 ± 5.2 years; 9 males). Participant eligibility included self-reported habitual sleep durations ranging from 7 to 9 h, with sleep onset between 22:00 and 01:00 and wake onset between 06:00 and 09:00. Participants were healthy younger and older adults who (a) did not nap more than twice a week; (b) had no history of medical, psychiatric, or sleep conditions; (c) were free of medications; (d) did not have any visual impairments or eye conditions not corrected by lenses; (e) were non-smokers; (f) were estimated to consumed less than 300 mg of caffeine per day and less than 14 standard alcoholic drinks per week; (g) had not worked shift work or traveled across two time zones in the past 3 months; (h) were not extreme morning or evening types according to the Morningness–Eveningness Questionnaire [30]; and (i) reported a BMI ≥ 18 and ≤ 30. Female participants were not currently pregnant, breastfeeding, or using hormonal contraception. Participants were good, healthy sleepers as indicated by Epworth Sleepiness Scale (< 11) [31]; Insomnia Severity Index (< 8) [32]; Fatigue Severity Scale (< 36) [33]; Pittsburgh Sleep Quality Index (< 6) [34]; and low risk for OSA as indicated by the Berlin [35] and STOP-BANG [36] and of good general mental/physical health as indicated by the Patient Health Questionnaire PHQ-9 (< 5) [37]; Generalized Anxiety Disorder GAD-7 (< 5) [38]; and Depression Anxiety Stress Scale (depression ≤ 4; anxiety ≤ 3; stress ≤ 7) [39]. All participants underwent psychological assessment using the Structured Clinical Interview for DSM-4 [40] to exclude any individual with any history (or close family relative) with an Axis-1 psychiatric illness, had an ECG to ensure no undiagnosed cardiac abnormalities, and completed one night of at-home respiratory monitoring using ApneaLink (ResMed Corporation, Poway, CA, USA) to screen for obstructive sleep apnoea (defined as an Oxygen Desaturation Index [ODI] 4% > 5). Older adults also underwent neuropsychological assessment to ensure they did not have any neurocognitive impairment, defined as scores ≤ 1.5 standard deviation (SD) below age-matched norms on the California Verbal Learning Test [41] and Comprehensive Trail Making Test [42], and had a Mini-Mental State Examination score of ≤ 24 [43].

The study was approved by the Monash University Human Research Ethics Committee (approval number 9215). Written informed consent was provided by participants and they were reimbursed up to $675 AUD for their time.

### Study protocol

**Figure 1.**
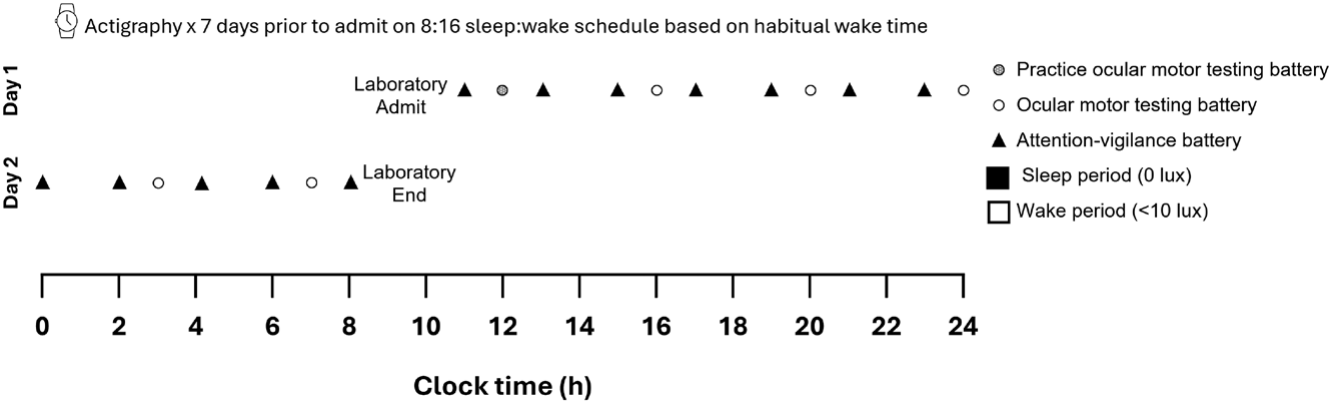
In-lab study protocol: The at-home sleep period (black bars) comprised of 8 h time in bed, while the wake period (white bars) represents 26 h of continued wakefulness. The timing of the tests is shown, including the ocular motor tasks (pro- and anti-saccade) for both the practice test (lighter grey circle) and those utilized in analyses (open circle), and the attention-vigilance battery (black filled triangle), which included the PVT and KDT, with simultaneous EEG.

The study protocol is shown in Figure 1. To ensure all participants were well-rested prior to admission, all participants kept a fixed sleep and wake schedule at home for 1 week, which included an 8-h sleep opportunity, and was determined based on their self-reported sleep times [44]. Compliance was monitored via actigraphy (Actiwatch-2, Philips Respironics, USA) and sleep diaries with time-stamped call-ins at bed and wake time. Participants abstained from caffeine, alcohol, nicotine, medications (both prescription or over-the-counter), and recreational drugs for at least 24-h prior to admission to the laboratory and throughout the study. This was confirmed by urine toxicology and breathalyzer upon arrival. Participants were admitted to the laboratory 2-h post-wake and underwent a 26-h period of extended wakefulness in a time-isolated and sound-attenuated testing suite, and were continually monitored for wakefulness by research staff. Light levels were maintained < 10 lux, and temperature was maintained at 21°C ± 1°C. Participants were provided with regular meals (breakfast, lunch, dinner, and snacks) and had water ad lib. Administration of the testing batteries began 4-h post-wake, and the test battery was repeated two-hourly or four-hourly (see below for further explanation and Figure 1), with the final task completed at 26-h post-wake. Between testing, participants could move around the suite and engage in light activities such as reading, playing board games with research staff, or watching (non-live) television.

### Test battery

The test battery was designed to capture a wide range of tasks typically employed in sleep research that capture a variety of behavioral and physiological responses to sleep deprivation. The test battery included the bi-hourly administration of the PVT and Karolinska drowsiness task (KDT) with simultaneous EEG/EOG and an ocular motor assessment of attention (reflexive, inhibitory control) administered four-hourly. Due to our hypothesis that younger adults are more likely to fall asleep during sleep deprivation, while older adults may be vulnerable to executive components of attention, we categorized the outcomes from these four modalities/tasks into: those reflecting a transition to, or state of, sleep, termed “*sleep initiation”* outcomes (e.g. EEG microsleep, SEMs, EEG delta power, and long PVT lapses [> 3 s]), and those that were not wholly explained by the tendency to fall asleep, termed “*non-sleep initiation”* outcomes (e.g. PVT Mean RT/Lapses, reflexive attention, and inhibitory control)—see Table 2.

**Table 2.** Effect of sleep deprivation (TSW) and age across tasks. Descriptives (mean, SD) and inferential statistics (coefficient, CIs) for sleep-initiation and non-sleep-initiation outcomes

	Task	Metric	Well-rested		Sleep-deprived		TSW		Age		TSW × Age	
			Younger	Older	Younger	Older	*F*/χ^2^ [CI]	*p*	*F*/χ^2^ [CI]	*p*	*F*/χ^2^ [CI]	*p*
**Sleep initiation**	**EEG**	EEG microsleeps (#)	0.04 (0.03)	0.01 (0.01)	4.4 (1.9)	1.1 (0.5)	110.0 [5]	**<.0001**	6.4 [1]	**.011**	24.1 [5]	**<.0001**
		EEG SEMs (#)	0.5 (0.2)	0.1 (0.1)	10.7 (3.0)	4.9 (1.8)	387.0 [1]	**<.0001**	6.3 [1]	**.012**	38.9 [5]	**<.0001**
		Delta power (%)	27.5 (11.3)	18.7 (7.0)	38.1 (9.7)	24.2 (9.3)	10.2 [1,74.4]	**<.001**	15.3 [1,32.6]	**<.001**	2.6 [5, 74.4]	**.031**
	**PVT**	Behavioral microsleep (Lapses > 3 s) (#)*	0.1 (0.1)	0.03 (0.01)	4.4 (1.3)	1.9 (0.6)	170.0 [5]	**<.0001**	4.5 [1]	**.034**	6.5 [5]	.259
**Non-sleep initiation**	**PVT**	Mean RT < 500 (ms)	288.0 (22.9)	304.3 (24.0)	328.0 (29.4)	346.4 (35.5)	30.9 [5, 97.7]	**<.001**	2.9 [1,33.0]	.096	1.9 [5, 87.1]	.107
		Lapses 500–2999ms (#)	2.1 (0.6)	1.7 (0.4)	12.8 (2.0)	13.0 (2.3)	29.5 [5, 69.2]	**<.001**	0.00 [1, 34.2]	.988	0.85 [5, 69.2]	.519
	**Ocular motor**	Pro-saccade latency (ms)	164.6 (18.4)	195.7 (24.4)	168.8 (19.0)	198.8 (18.5)	6.5 [3, 80.0]	**<.001**	22.8 [1,32.3]	**<.001**	0.97 [3, 80.3]	.409
		Anti-saccade inhibitory errors (%)	22.0 (21.0)	43.7 (23.8)	30.6 (20.2)	52.7 (21.3)	16.1 [3, 86.0]	**<.001**	10.3 [1,31.9]	**.003**	1.7 [3, 86.0]	.170

*Note:* Outcomes presented as mean (SD) and statistics presented as coefficient [95% CI]. SEMs = slow eye movements; TSW = time since wake; CI = confidence intervals; well-rested ≤ TSW16; sleep-deprived ≥ TSW17.

#### Psychomotor vigilance task (PVT)

The PVT is a 10-min task of sustained attention [45]. Participants monitored a rectangle in the center of the screen and responded as quickly as possible to an ascending millisecond stopwatch appearing at random intervals (2–10 s). Participants’ reaction times (RT) were displayed on the screen prior to the next trial commencing. Failure to respond within 10 s resulted in an audible tone (~75 dB), and button presses in the absence of the timer resulted in visual feedback of “error”.

Several outcomes for the PVT were measured in line with our hypotheses. For *sleep initiation* outcomes, we calculated the number of PVT lapses more than 3000 ms. We term these ‘behavioral microsleeps’ as they are 95% likely to occur with the eyes being closed [46] and have been used previously to highlight individual vulnerability to sleep loss [47]. For *non-sleep initiation* outcomes, we used mean RT of timely responses (< 500 ms), as these responses are also sensitive to sleep loss but are more than 95% likely to occur with the eyes open [46]. In addition, traditional metrics from the PVT (mean RT for all RT, all PVT lapses > 500 ms, and performance variability (RT SD)) are reported for comparison.

#### Electroencephalography/electrooculography

Electroencephalography (EEG) and electrooculography (EOG) were recorded continuously using Brainvision Recorder using ACTi-Cap Slim 32 active channels based on the 10:20 international system, with a Live Amp compact wireless portable amplifier (Brain Products, Munich, Germany). Data were sampled at 512 Hz, filtered between 0.3 and 30 Hz, and a Notch filter at 50 Hz. Channels were re-referenced to Tp9 and Tp10 for visual scoring of EEG data. EEG was captured both while engaging in a task (during the PVT), and while sitting quietly (during a Karolinska drowsiness test).

##### EEG microsleep/EOG slow eye movements

*Sleep initiation* outcomes included a number of microsleeps and SEMs recorded during the PVT as this more closely reflects working environments (i.e. the capacity to remain awake while engaged in a task). A microsleep was defined as an intrusion of theta or delta activity greater than 3 s in the absence of any artifacts or eye blinks across all available channels, and SEMs were scored as the EOG trace deviating and return to baseline in both EOG channels [48]. Both events were visually scored, and verified, by an experienced scorer.

##### EEG delta power

Spectral analyses of EEG data were derived from the KDT which required participants to sit still with eyes closed for 2 min [49]. This ensures a “clean” segment of EEG free from the ocular artifact. For power spectral analysis, all channels were re-referenced to an average reference across all channels using Brainvision Analyser (Brain Products, Munich, Germany). Total power spectra (μV^2^) for the alpha (8–12 Hz), theta (4–8 Hz), and delta (1–4 Hz) bandwidths were estimated for C3 (substituted for C4 when unavailable) in 2 s epochs using a 0.5 frequency resolution and Hanning window (5% window length; periodic). To control for individual differences, relative power density was calculated for each bandwidth as the relative percentage of the total power spectra (1–30 Hz). Delta (1–4 Hz) was used as the primary metric to reflect *sleep initiation*. Importantly, this bandwidth is correlated with microsleeps in general [50], but also the transition “in” and “out” of the microsleep indicating the brain has transitioned to a sleep state [51]. Alpha and theta are also reported for comparison.

#### Ocular motor attentional control paradigms

In a visually rich environment, attention can be allocated in a goal-oriented manner, where attention is allocated “top-down” based on internal demands, or in a reflexive manner where attention is allocated based on the external capturing of attention (bottom-up, stimulus-driven) [52, 53]. These two aspects of attention allocation can be captured using two well-established ocular motor paradigms: the pro-saccade and anti-saccade [54, 55]. These have been used previously in both sleep restriction [56] and sleep deprivation [57]. Importantly, they examine attention allocation in the absence of sleep transition events as only saccades that occurred in the absence of eye closures and/or eye rolls are analyzed [56, 57]. Outcomes from these tasks are thus used to form “*non-sleep-initiation”* outcomes. This test battery occurred at four-hourly intervals, beginning 5 h post-wake. Task order was randomized and counterbalanced between test sessions.

##### Pro-saccade task (reflexive attention)

This task mimics the Psychomotor Vigilance Task in that both tasks require a response to a single recurring stimulus in the absence of any external conflicting demands on the attention system. This task however allows us to look at reflexive attention in the absence of ocular indices of sleepiness, to assess specific impairments in reflexive attention. After staring at central fixation for a random duration of 1000, 1250, or 1500 ms, the pro-saccade task requires participants to initiate a reflexive saccade toward the peripheral stimulus. One block of 24 trials was presented, for a total task duration of approximately 73 s. Mean response latency was calculated as the time between the appearance of the peripheral stimulus and the initiation of a saccade toward it.

##### Anti-saccade task (inhibitory control)

The anti-saccade task requires participants to inhibit a reflexive saccade toward a peripheral stimulus and instead look in the mirror opposite location [54]. This peripheral stimulus remained on screen for a random duration of 1250 or 1600 ms before the next trial began with central fixation (which remained on screen for 1500 ms). Two successive blocks of 24 trials were presented, with a short break in between of approximately 5–7 s, for a total task duration approximating 180 s. Percentage of inhibitory errors (defined as the percentage of valid trials where participants looked toward the stimulus instead of the mirror location) was calculated.

During the ocular motor tasks, participants’ eye movements were recorded via monocular tracking using an Eyelink 1000 Plus eye tracker (SR Research Ltd, Ontario, Canada) at a sampling rate of 1000 Hz. The stimulus used was consistent between tasks and was comprised of a green cross (subtending 1.6 × 1.6°C of the visual angle) against a black background. Each trial consisted of a no-gap paradigm, whereby stimulus presentation began in the center of the screen and for each trial, the stimulus then immediately appeared in a peripheral location, with no overlay. The stimulus then disappeared at the peripheral location and simultaneously reappeared at central fixation in preparation for the next trial. Each presentation of the peripheral stimulus occurred at random but equal frequency at four different locations, either 5° or 10° to the left or right of central fixation.

#### Task outcomes: sleep initiation versus non-sleep initiation

In summary, the outcomes from the four tasks/modalities are categorized into *sleep-initiation* and *non-sleep-initiation* metrics, as seen in Table 2.

Sleep-initiation outcomes refer to measures reflecting a transition to, or state of, sleep, and include EEG microsleeps and SEMs recorded during the PVT, relative EEG delta power (1–4 Hz) during the KDT, and long PVT lapses (> 3000 ms) which have previously been associated with a high (95%) probability of eye closure [46].

Non-sleep-initiation outcomes refer to measures not wholly explained by the process of falling asleep. These include mean reaction time for timely responses on the PVT (average speed for RTs 100–500 ms), number of PVT lapses between 500 and 2999 ms (i.e. excluding long lapses), speed of reflexive attention (saccade latency) on the pro-saccade task, and inhibitory errors on the anti-saccade tasks.

#### Data processing and statistical analyses

For PVT, RTs < 100ms were removed (due to anticipation). For spectral analysis, EEG data were visually examined and 2 s epochs with artifact (e.g. blinks) were removed. Any KDT recordings with < 60 s of clean data were excluded from the analysis. Ocular motor data were analyzed using a customized MATLAB program (MATLAB, The Mathworks Inc., Natick, MA). Saccades were semi-automatically detected and visually confirmed, defined as movements greater than 1.5°. As we were interested in attentional allocation in the absence of sleep events, trials that included ocular indices of sleepiness, such as eye closures or blinks, were excluded (as per [56, 57]). These trials were defined as events where the participant’s eye was closed or not within 2.5° of central fixation upon stimulus presentation; fixation was not stable at saccade onset (greater than 1.5° movement); or when participants blinked or closed their eye between stimulus presentation and the completion of their saccade response. Response latencies below 100 ms were also removed as these are considered anticipatory [56]. Data from an ocular motor session were excluded if more than 60% of trials were removed.

##### Missing data

In total, 408 testing sessions were completed (12 sessions × 34 participants). PVT data was available for all test sessions. Of 408 EEG/EOG recordings during the PVT, data were available for *n* = 394 microsleep assessments (3.4% data loss) and *n* = 343 SEM assessments (15.9% loss). Data loss was due to excess artifact in necessary channels (*n* = 2 sessions), recording or equipment malfunctions (*n* = 12 sessions), and loss of EOG signal during recording (*n* = 51 sessions). For EEG/KDT recordings, spectral data was available for *n* = 374 (8.3% data loss) recordings. Data loss was due to noise in both C3 and C4 (*n* = 19 sessions) and recording or equipment malfunctions (*n* = 15 sessions).

Due to the unfamiliarity of the ocular motor tasks and to mitigate any chance of practice effects [58], the first session (hour 5) familiarized participants with the tasks and was not included in the final dataset. Of a possible 170 testing sessions (5 sessions × 34 participants) for the pro- and anti-saccade tasks, a total of three anti-saccade and four pro-saccade sessions were lost due to protocol adjustment (participant nausea) or technological difficulties (1.8% and 2.3% data loss, respectively). From these sessions, data were cleaned for quality controls (e.g. > 60% of trials with eye closure/rolling removed), resulting in the removal of 10 anti-saccade test sessions (*n* = 8 younger group, *n* = 2 older group; 9.4% and 2.4% loss, respectively) and 16 pro-saccade test sessions (*n* = 11 younger group, *n* = 5 older group; 12.9% and 6.2% loss respectively). The final dataset therefore comprised 157 anti-saccade test sessions (*n* = 77 younger group, *n* = 80 older group) and 150 pro-saccade sessions (*n* = 74 younger group, *n* = 76 older group).

### Statistical analysis

**Figure 2.**
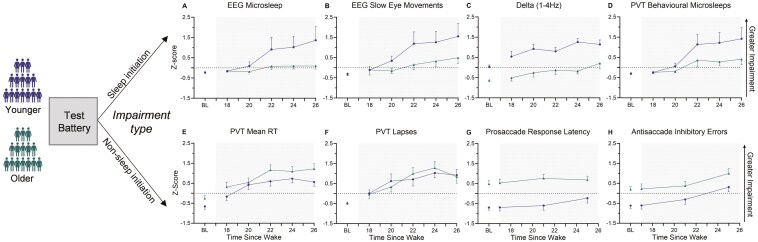
Age-related changes in response to sleep deprivation. Changes in sleep initiation-based metrics (A–D) and non-sleep-initiation-based metrics (E–H) as a function of increasing sleep deprivation for younger and older adults. All outcomes represent group mean (SE) of *z*-scores. Dotted lines represent the 0-point (no deviation from the mean of distribution: all participants/time points), with shading indicating sleep deprived hours (> 17 h). The unshaded normal waking day (< 16 h) has been averaged and presented as the baseline (BL).


For all outcomes, data from the first 16 h were averaged to form a baseline measurement of normal waking performance [48, 59]. The effect of sleep deprivation and age on EEG metrics (relative spectral power for theta and delta activity), PVT outcomes (mean RT, lapses, and performance variability), and ocular motor outcomes (anti-saccade task: inhibitory errors; pro-saccade latency) were assessed using linear mixed model analysis (SPSS v28). Reaction time and latency for all cognitive tests were normalized by calculating the reciprocal [1/(×/1,000)], while the number of lapses (> 500 ms) was transformed using [(√ *n*) + (√ *n* + 1)] [60]. The proportion of inhibitory errors on the anti-saccade task was normalized using an arcsine square root transformation. Time since wake (TSW) and age were modeled as fixed factors and participant was modeled as a random factor. The covariance structure that provided the lowest Schwarz’s Bayesian information criterion value for each mixed model was used [61]. Due to the large number of zeros and overdispersion of data our initial steps to conduct linear mixed model analysis for behavioral microsleeps (from the PVT), EEG microsleeps, and SEMs were unsuccessful due to non-convergence despite attempts to resolve (e.g. increasing the number of step halvings). As a robust alternative, these three variables were analyzed using repeated-measures negative binomial regression (generalized estimating equations with a log-link function) [62]. Time since wake (TSW) and age were modeled as fixed factors and the participant was modeled as a random factor. An auto-regressive working correlation structure was utilized for each, with model-based estimators. All significant interaction terms were followed up with pairwise comparisons of the estimated marginal means and were corrected with a false-discovery rate adjustment to account for type 1 error [63], with *p*_adj_ reported for all post-hoc tests. For standardization of outcomes in Figure 2 only, all values were converted to *z*-scores:

$$\begin{array}{l} z=\frac{X-u}{\sigma } \end{array}$$

Where μ and 𝜎 represent the mean and standard deviation, respectively, calculated across all participants and time points, to facilitate comparison between groups.

To compare the magnitude of impairment across each outcome for both age groups, mean change scores were calculated, defined as the difference between the average of the baseline hours (≤ 16 h) and the average of the sleep-deprived hours (≥ 17 h). Effect sizes (Cohen’s *d*) were calculated for between-groups comparisons, where the mean difference was divided by the pooled standard deviation. To examine inter-individual differences in sleep deprivation response, these change scores (sleep deprivation—well-rested) were examined on a person-by-person basis. As a secondary outcome, we explored any potential influence of sex. While our study was underpowered to assess any sex-related effect, we have presented the mean change scores for each outcome by age and sex with effect sizes using a correction for small sample (*n* < 20) bias (Hedges’ *g*_*s*_ [64]).

## Results

Data were collected from 34 participants across 26 h of sleep deprivation. Demographic and sleep outcomes are shown in Table 1. The distribution of males and females is approximately equal in both groups, with older adults exhibiting a higher ODI 4% (*p* = .035) and a trend toward greater morning preference (*p* = .059). No differences in subjective sleep quality, daytime sleepiness, pre-study total sleep time, or sleep efficiency were observed (*p* > .070).

**Table 1.** Demographics and sleep outcomes for younger and older adults

Outcomes	Younger	Older	Significance values (*p*)
**Demographics**			
Age (years)*	24.5 (3.2)	57.3 (5.2)	**<.0001**
Sex (M:F)	10:7	9:8	.730
Body mass index (kg/m^2^)	23.6 (3.2)	24.4 (2.7)	.428
**Sleep screening**			
ESS (/24)	3.6 (2.0)	2.8 (2.0)	.271
PSQI (/21)	2.5 (1.6)	2.5 (1.7)	1.000
ISI (/63)	1.4 (1.4)	1.8 (1.8)	.393
MEQ (/86)	38.6 (5.2)	42.1 (5.0)	.059
ODI 4% (events/h)	0.5 (0.5)	1.3 (1.5)	**.035**
**Sleep actigraphy monitoring**			
Time in bed (h)	8.1 (0.1)	8.1 (0.1)	.949
Total sleep time (h)	6.7 (0.6)	6.3 (0.7)	.460
WASO (min)	55.3 (25.6)	75.9 (35.5)	.070
SOL (min)	13.6 (10.4)	16.8 (28.1)	.673
Sleep efficiency (%)	83.4 (7.6)	78.1 (8.6)	.072

*Note:* Each outcome presented as mean (SD) unless otherwise stated. ESS = Epworth Sleepiness Scale; PSQI = Pittsburgh Sleep Quality Index; ISI = Insomnia Severity Index; MEQ = Morningness–Eveningness Questionnaire (short version); WASO = wake after sleep onset; SOL = Sleep onset latency.

### Sleep-initiation metrics

#### EEG physiological microsleeps

A total of 469 microsleeps were detected across the protocol. Of these, 463 were observed during sleep deprivation (TSW 18 to 26) from 19 out of 34 (56%) individuals: 53% of all younger adults (9/17) and 59% of all older adults (10/17). There was a significant age × TSW interaction (χ^2^(5) = 24.06, *p* < .0001), such that younger adults experienced more microsleeps during sleep deprivation relative to baseline (for all time points TSW 18 to 26 *p*_adj_ < 0.039). This was similar for older adults (TSW 18 to 26 *p*_adj_ < 0.04), except TSW 20 did not significantly differ from baseline (being just above the acceptable level of significance [*p*_adj_ = 0.058]). During sleep deprivation, more EEG microsleep were observed in younger adults compared to older adults after 20 h awake (TSW 20 to 26, *p*_adj_ < 0.016).

There were main effects of TSW and age, such that microsleeps were higher during sleep deprivation (χ^2^(5) = 110.03, *p* ≤ .0001) and younger adults had more microsleeps compared to older adults overall (χ^2^(1) = 6.39, *p* = .011). This can be seen in Figure 2A and is shown in Table 2.

#### Slow eye movements (SEMs)

A total of 1154 SEMs were detected across the protocol. Of these, 1092 were observed during sleep deprivation (TSW 18 to 26) from 22 out of 34 individuals. This proportion was balanced across age groups (11/17, 64.7%, for both younger and older adults). There was a significant age × TSW interaction (χ^2^(5) = 38.90, *p* ≤ .001) that exhibited a similar profile to EEG microsleeps. Here, both younger and older adults had more SEMs during sleep deprivation compared to baseline (for all time points, younger *p*_adj_ < .003; older *p*_adj_ < .002). During sleep deprivation, the younger group had a trend toward more SEMs compared to older adults after 20 h awake (TSW 20 to 26, *p*_adj_ = .055 to .097). No differences were observed for TSW 18 (*p*_adj_ = .800).

There was a main effect of TSW (χ^2^(5) = 386.95, *p* ≤ .001) and age (χ^2^ (1) = 6.35, *p* = .012), such that SEMs increased with sleep deprivation, and young adults had more SEMs compared to older adults overall (see Figure 2B, Table 2).

#### EEG delta power

There was a significant age × TSW interaction in average power spectra in the delta (1–4 Hz) bandwidth (*F*_5,74.44_ = 2.61, *p* = .03). Here, younger adults had greater relative delta power during sleep deprivation compared to baseline (for all time points, *p*_adj_ < .0008), while older adults also exhibited greater power but significant differences emerged only after 20 h awake (*p*_adj_ < .004). Greater delta power was observed in younger adults compared to older adults for baseline (*p*_adj_ < .047) and sleep deprivation periods (all TSW *p*_adj_ < .009).

There was also a main effect of both TSW (*F*_5,74.44_ = 10.17, *p* < .001) and age (*F*_1,32.57_ = 15.31, *p* < .0001) (see Figure 2C, Table 2).

As a comparison to typical power spectra outcomes, we observed no age × TSW interaction for the theta band (4–8 Hz; *p *= .269) or alpha band (8–12 Hz; *p *= .101). While both power spectra increased following sleep deprivation (theta: *F*_5,106_._57_ = 7.01, *p* < .001; alpha: *F*_5,142.76_ = 11.49, *p* < .001), no main effects of age were observed (theta: *p *= .437, alpha: *p *= .101).

#### PVT behavioral microsleeps (responses > 3000 ms)

A total of 547 behavioral microsleeps were detected across the protocol. Of these, 535 were observed during sleep deprivation (TSW 18 to 26) from 19 out of 34 (55.9%) individuals. These included 59% of all younger adults (10/17) and 53% of all older adults (9/17). While there was no significant interaction between age and TSW (*p* = .259), there was a main effect of TSW (χ^2^(5) = 170.05, *p* < .0001) and age (χ^2^ (1) = 4.52, *p* = .034). Here, behavioral microsleeps increased due to sleep deprivation from TSW 18 onward (*p*_adj_ < .009), and younger adults had more behavioral microsleeps relative to older adults overall (see Figure 2D, Table 2).

### Non-sleep-initiation metrics

#### PVT mean RT

Mean RT of timely responses (< 500 ms) was first examined to assess sustained attention in the absence of suspected sleep-initiating mechanisms (e.g. increased probability of eye closures). No interaction between age and TSW was observed (*p* = .107). While mean RT became slower overall with TSW (*F*_5,87.71_ = 30.896, *p* < .001), there was no main effect of age (*p* = 0.096). For TSW, sleep deprivation effects were observed from TSW 18 onward (*p*_adj_ < .001) (see Figure 2E, Table 2).

#### PVT lapses (responses 500–2999 ms)

Similarly, for PVT lapses excluding long behavioral microsleep lapses, no interaction between age and TSW was observed (*p* = .519), as well as no main effect of age (*p* = .988). These PVT lapses (500–2999 ms) did increase with TSW (*F*_5,69.16_ = 29.52, *p* < .001) as expected, with an increase from TSW 18 onward (*p*_adj_ < .001) (see Figure 2F, Table 2).

Common PVT metrics are available and shown in Supplementary Data S1.

#### Prosaccade latency

No interaction between age and TSW was observed (*p* = .409) and the mean change in response latency was small for both age groups (7.0 vs. 4.1 ms). There was a main effect of age (*F*_1,32.32_ = 22.81, *p* < .001) and TSW (*F*_3,80.03_ = 6.48, *p* = .001), such that older adults were slower to respond to the stimulus and sleep deprivation slowed responses overall. For TSW, sleep deprivation effects were observed from TSW 21 onward (*p*_adj_ < .008) (see Figure 2G, Table 2).

#### Anti-saccade inhibitory errors

There was no significant age × TSW interaction (*p* = .170), although inhibitory errors increased with TSW (*F*_3,85.98_ = 16.09, *p* < .001) and older adults had a greater number of inhibitory errors compared to younger adults overall (*F*_1,31.90_ = 10.31, *p *= .003). For TSW, sleep deprivation effects were observed at TSW 25 only (*p*_adj_ < .001) (see Figure 2H, Table 2).

### Task-dependent age-related responses to sleep deprivation (effect sizes)

**Figure 3.**
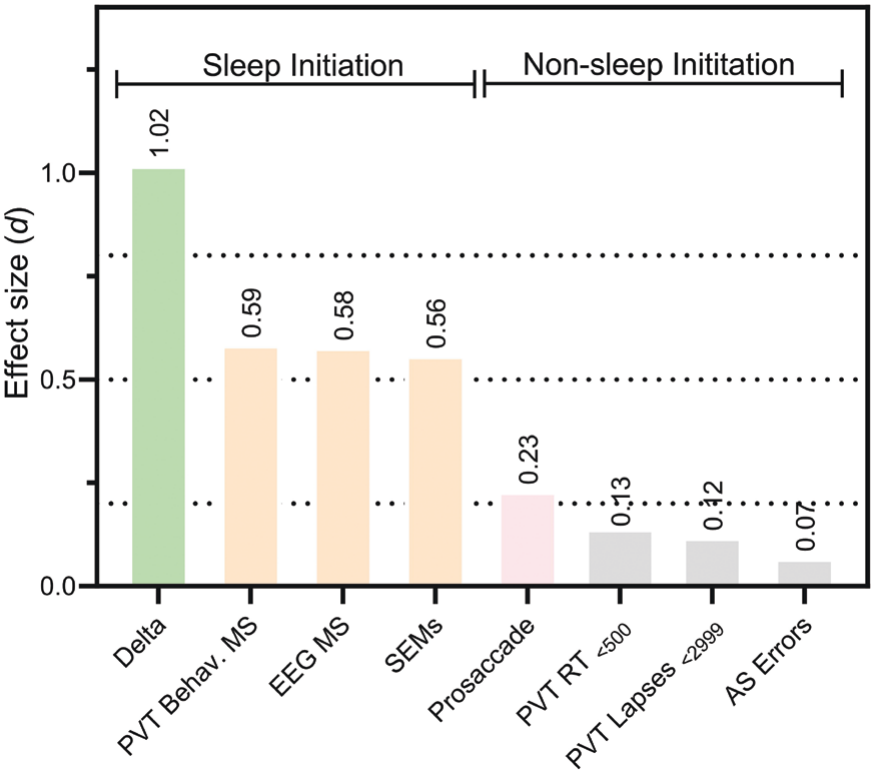
Effect sizes (Cohen’s d) for the impact of sleep deprivation on outcomes between groups. Average performance when well-rested hours (≤ 16 h) relative to sleep-deprived hours (≥ 17 h). Horizontal dotted lines indicate cut-offs for negligible effects (d<0.2), small (d = 0.02 - 0.5)), medium (d = 0.05 - 0.8), and large (d > 0.8) effect sizes. Plotted in order of magnitude from left to right these are: [sleep initiation metrics] relative delta power spectra, PVT behavioral microsleeps (RTs > 3000 ms), EEG microsleeps, slow eye movements (SEMs), and [non-sleep initiation metrics] pro-saccade latency, PVT mean RT (< 500 ms), PVT lapses (500–2999 ms), and anti-saccade errors.

To more directly examine any task-dependent effects of sleep deprivation between age groups, we calculated effect sizes for change scores (averages for sleep deprivation—well rested) of all task outcomes and compared younger versus older adults. As seen in Figure 3 we found larger effect sizes for those metrics that reflected sleep initiation outcomes, including a large effect size for delta power (*d* = 1.02) and medium-to-large effect sizes for EEG microsleep, behavioral microsleep and SEMS (*d* > 0.56). Only small to medium effect sizes were observed for all non-sleep initiation metrics (*d* < 0.23).

### Inter-individual differences

**Figure 4.**
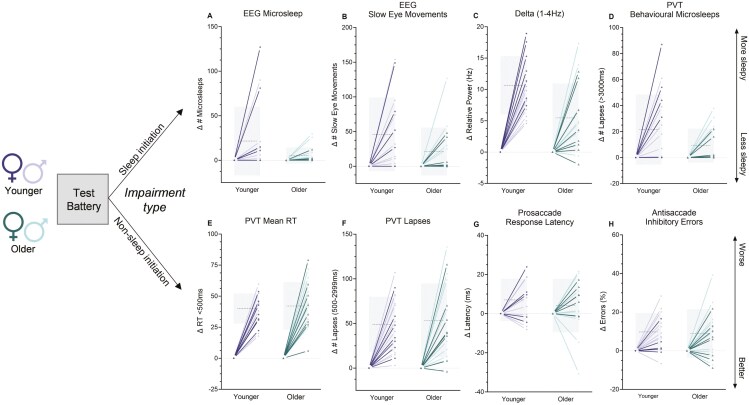
Individual differences in the response to sleep loss for younger and older adults. Data represent the mean change score for the average of the well-rested hours (≤ 16 h awake) compared to the average of sleep-deprived hours (≥ 17 h) for performance-based metrics (A–D) and sleep-initiation-based metrics (E-–H). All outcomes include individual datapoints, with group mean (dotted line) and 1SD (± as shading). Darker lines represent females and lighter lines represent males in either age group.

To examine inter-individual differences in sleep deprivation response, change scores were calculated (sleep deprivation—well-rested) for each individual for all outcomes (see Figure 4).

#### Sleep initiation metrics

For EEG microsleeps, variability in the response to sleep loss (change score) was larger within the younger group (range 0 to 127 total, mean 21.6 ± 38.6) compared to the older group (range 0 to 30 total, mean 5.3 ± 9.2, Figure 4A). This was similar for SEMs where response variability was greater for younger adults (range 0 to 154, mean 43.2 ± 52.7) than older adults (range −1 to 127, mean 21.2 ± 34.6, Figure 4B). For relative delta power spectra, the younger and older group had comparable variability (younger 4.6%–18.9%, older −1.98%–17.34%) although the mean change score was higher for the younger (10.65 ± 4.7%) versus older (5.4 ± 5.5%) groups (Figure 4C). PVT behavioral microsleeps also revealed large differences in within-group variability for younger (range 0 to 87, mean 48.6 ± 27.0) and older adults (range 0 to 38, mean 22.2 ± 13.1, Figure 4D).

#### Non-sleep initiation metrics

When examining metrics that were not specifically focused on sleep initiation, variability within the groups was more comparable. This includes PVT Mean RT of timely responses (younger: range 17.9 to 59.7, average±SD 40.0 ± 12.2; older: 6.0 to 79.0, 42.07 ± 19.3) and PVT lapses excluding long lapses more than 3000 m (younger: 3 to 107, 49.1 ± 30.9; older: −4.0 to 136.0, 53.4 ± 41.5), pro-saccadic latency (younger: −8.2 to 23.9, 7.0 ± 10.8; older: −30.5 to 21.6, 4.1 ± 13.7) and anti-saccade errors (younger: −6.3 to 28.4, 9,7 ± 9.8; older: −8.9 to 39.3, 8.9 ± 12.6) (see Figure 4E–H).

Variability in common PVT metrics used in prior aging-sleep deprivation studies is shown in Supplementary Data S2.

#### Individual differences—effect of sex

**Figure 5.**
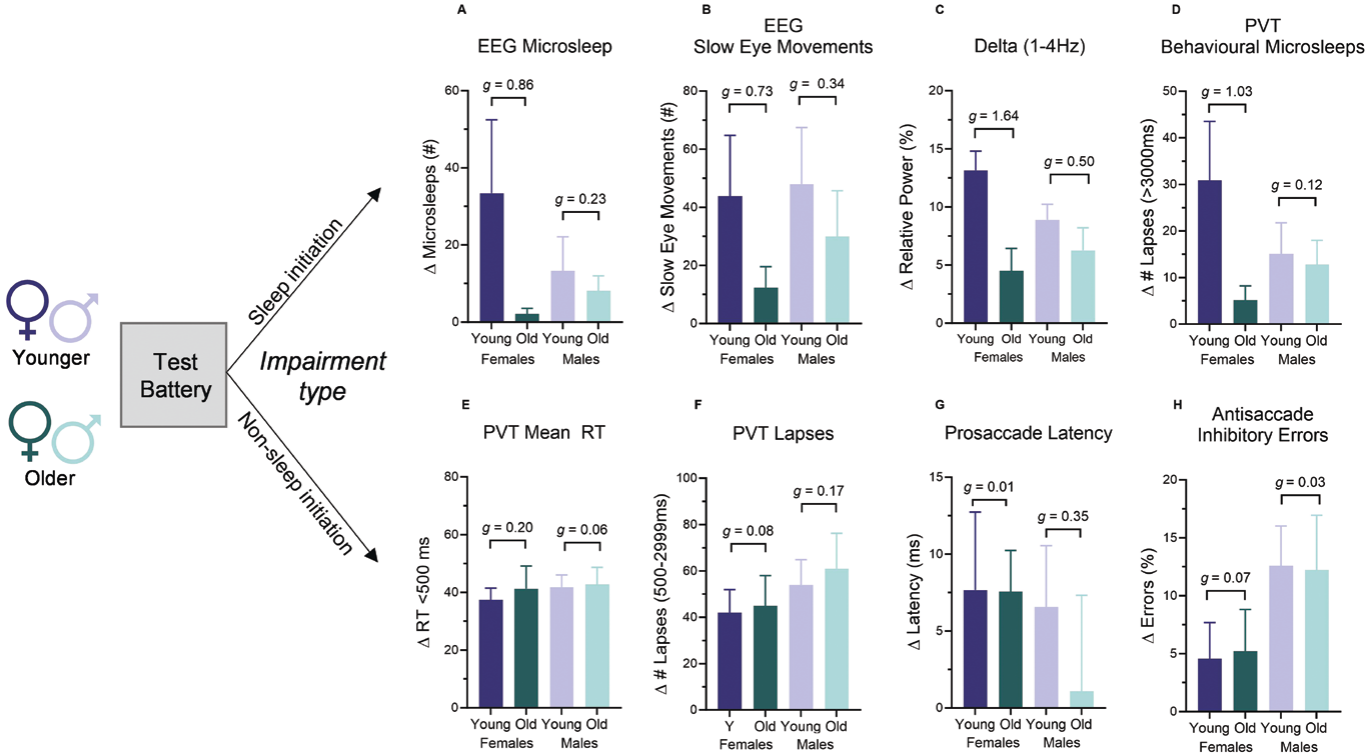
Mean change by age and sex following sleep deprivation. Data represent the change score from the well-rested hours (≤ 16 h awake) compared to the sleep-deprived hours (≥ 17 h). All outcomes are presented as mean (SEM), with effect sizes Hedges’ *g*).

Due to prior evidence of enhanced vulnerability to sleep loss for females relative to males, we explored any potential effects of sex with respect to individual differences observed within the younger and older groups. In our exploratory observations, younger females appear to be more vulnerable compared to older females for EEG microsleeps (*g* = 0.86), number of SEMs (*g* = 0.73), relative delta power (*g* = 1.64), and number of behavioral microsleeps (*g* = 1.03). This was not observed for non-sleep initiation metrics (*g* < 0.20), nor for males for any metrics (*g* < 0.50) (see Figure 5).

## Discussion

Our study examined task-dependent effects in the age-related response to total sleep deprivation, culminating in three main observations. First, we emphasize that older adults were vulnerable to sleep deprivation when compared to their well-rested state. Second, task-dependent effects were apparent, such that younger individuals appear more vulnerable on tasks that reflect sleep initiation (e.g. microsleep, SEM, and long lapses), compared to the older group. Third, these group-level vulnerabilities to sleep initiation (for younger adults) appear to be driven by a limited number of vulnerable individuals. Our work suggests that any study exploring vulnerability to sleep loss across the lifespan should examine performance across multiple tasks (due to task dependency) and at the individual level (due to individual differences) to better understand and predict vulnerability to sleep loss in real-world settings.

Sleep deprivation impaired performance across all metrics as expected [4]. While we confirm previous findings that older adults better tolerate sleep deprivation relative to younger adults [15], our data suggest that this may be task/outcome dependent. Specifically, younger adults appeared more vulnerable to falling asleep during sleep deprivation as reflected by more EEG microsleep, SEMs, behavioral microsleep (responses > 3 s), and relative delta power compared to older adults. This specificity to sleep initiation events was supported by the lack of age-related differences in the response to sleep deprivation for the non-sleep-initiating-based tasks/metrics, including reflexive attention, inhibitory control, and PVT metrics where sleep-initiation trials were removed (i.e. trials associated with long eye closure and/or SEMs as previously described [46, 57]). As PVT lapses may relate to other aspects of impairment such as diverted attention/distraction [65] or simple eye blinks [46], parsing out metrics that are more or less likely to be associated with the process of falling asleep may be beneficial in understanding vulnerability to sleep deprivation.

Contrary to previous studies (e.g. [12, 15]), we found no effect of age on the response to sleep deprivation for traditional PVT metrics (at the group level). Instead, both of our age groups showed deleterious effects of sleep deprivation on these outcomes (with no interaction). We offer a couple of observations in this respect. First, this may be due to the different ages of the participants. As we focused on older adults of working age, our older participants were younger (50–65 years) than those previously studied (60–76 years), while the younger groups were of comparable age. However, we do not believe this causes our lack of interaction as our older group appeared slightly more impaired than previous studies (14.9 vs. ~ 10.2 PVT lapses), while our younger group appeared less impaired (17.2 vs. ~ 21.8 PVT lapses, on average during sleep deprivation) [15]. Second, differences could be due to methodological differences from previous studies including duration of sleep deprivation [12], sex differences (e.g. use of all men [12] or majority (73%) men [15]), differences in sample size/unequal samples [15], and different calculation of PVT metrics [12], such as variability (e.g. range [12] versus standard deviation).

Individual differences in the response to sleep deprivation are well established [25, 26]. We now build on this phenomenon with respect to age, such that between-subject variance in the response to sleep deprivation appears larger in younger adults relative to older adults specifically for sleep initiation outcomes. When attention outcomes were examined in the absence of fall-asleep events (e.g. using an ocular motor paradigm or modified PVT metrics), age-related differences in the responses to sleep deprivation were less apparent (see Figure 3). We therefore suggest that group-level age-related vulnerability to sleep initiation is caused not by a population-level difference in response to sleep deprivation, but by a limited number of particularly vulnerable younger individuals (as described previously [25]). This should be interpreted with the following caveats in mind however: First, our results may be a random sampling consequence, and second, they may be caused by a sampling bias, such that older adults who have more experience of not coping well with sleep deprivation (e.g. more likely to have experienced sleep deprivation through children, work, life events) may self-select to not take part in sleep deprivation studies. However, there may be a true mechanistic driver behind enhanced vulnerability in a proportion (third) of younger adults. In a post-study follow-up, we confirmed that at least 75% of our older female adults were peri- or post-menopausal (*n* = 2 undetermined). We therefore present the intriguing possibility that lesser tolerance to sleep loss in younger adults may be caused, in part, by hormonal differences in older adults. For instance, ovarian sex hormones play a role in temperature regulation [66], and this rhythm is related to the capacity to respond quickly [67]. Indeed, previous research has demonstrated greater susceptibility to sleep deprivation in younger females [47, 68], particularly in the capacity to remain awake and alert. Our data were consistent with these observations whereby younger women appeared to be more vulnerable compared to older women for sleep initiation events (with large effect sizes). This was not observed for non-sleep initiation metrics, nor for males across any performance outcome. As we did not measure sex hormones nor determine objectively whether our female participants had gone through menopause, this remains speculative and future research might examine this further.

To our knowledge, our study is the first to assess age-related differences in reflexive attention and inhibitory control following sleep deprivation. Attentional control (including “bottom-up” responses to a visual stimulus and “top-down” inhibitory control) was examined in the absence of sleep events (e.g. eye closure, eye-rolling), with older and younger adults equally impacted by sleep deprivation. While both groups exhibited reduced inhibitory control following sleep deprivation, consistent with previous studies in younger adults [57, 69], the percentage of inhibitory errors made by the younger group after one night without sleep (46%), was comparable to that observed in the older group when well-rested (44%). Inhibitory control relies on the integrity of the prefrontal cortex and frontoparietal attention networks [53, 70], which are most vulnerable to the natural effect of aging [18–20] and sleep deprivation [21, 22]. It is therefore unsurprising that sleep-deprived younger adults may respond behaviorally similarl to older adults when well-rested on tasks that rely on these networks. Indeed, the concept that sleep deprivation can serve as a model for healthy aging with respect to executive function was first suggested more than two decades ago [23].

Our data should be interpreted with several caveats in mind. First, while these results are generalizable to healthy older adults, they do not apply to older individuals with medical or sleep disorder diagnoses that impact sleep and general well-being [71]. Given that cognitive performance is compromised for those with certain sleep and medical diagnoses, it is important to understand the response to sleep deprivation in older adults with comorbidities. Second, hormone levels can vary widely both across the menstrual cycle [72] and in the peri-post menopausal period [73], and this may explain some of our findings with respect to individual differences. Third, while our sample size is sufficient for detecting group-level differences, it is small for examining individual differences. These should therefore be interpreted with some caution and are there to highlight the need for further work in relation to inter-individual differences in the response to sleep deprivation for younger and older adults.

Despite these limitations, our study has important implications for occupational risk, particularly in shift workers. Understanding what makes an individual vulnerable to sleep deprivation is critical to mitigating the serious consequences of sleep deprivation in the workplace and on the roads. For example, activities that require staying awake and alert for prolonged periods of time, such as highway driving at night would be riskier for younger individuals [6]. Indeed, younger drivers represent the highest risk category for fall-asleep crashes [74], and are more likely to fall asleep while driving [6]. For jobs that require inhibitory control, such as ignoring peripheral distractors for air traffic controllers or airport security screeners, older adults remain more vulnerable when rested and under conditions of sleep deprivation. As older adults remain in the workforce for longer [10] understanding the effects of sleep deprivation in these individuals is essential to maintaining optimal health and safety in the workplace. Future studies are needed with much larger sample sizes, and potential evaluation of what causes these individual differences (e.g. genetics, sex, etc). While total sleep deprivation can occur in night-shift working populations, sleep loss in all its forms poses a significant public health issue. Based on our current findings, future research should explore whether age- and task-specific vulnerabilities to total sleep loss are also observed under other more common forms of sleep loss, such as sleep fragmentation or restriction.

To summarize, over two decades ago, Van Dongen and colleagues presented two critical discoveries in our field: the response to sleep deprivation is highly task-dependent and exhibits large individual differences [25]. Our data show that these discoveries remain true for age-related differences in vulnerability to sleep deprivation. Younger adults appear to be specifically more vulnerable to falling asleep during sleep deprivation, and this is driven by a subset of individuals who exhibit excessive impairment, for which sex may be one potential factor. Future studies should examine (i) whether this observation is consistent in real-world working environments and (ii) elucidate “who” is most vulnerable to sleep deprivation, and “how” they are more vulnerable. This knowledge will help reduce the number of serious accidents and injuries on our roads and workplaces due to sleep deprivation.

## Supplementary Material

zsaf144_suppl_Supplementary_Figures

## Data Availability

The data that support the findings of this study are available from the corresponding author upon reasonable request.
