# Supplementary material for: Age-related vulnerability to sleep deprivation is task dependent and influenced by large inter-individual differences in younger adults
Source: Sleep. 2025 May 30;48(10):zsaf144. doi: 10.1093/sleep/zsaf144 (PMC12515600; doi:10.1093/sleep/zsaf144)
Supplement: zsaf144_suppl_Supplementary_Figures [file zsaf144_suppl_supplementary_figures.docx]

**Age-Related Vulnerability to Sleep Deprivation is Task Dependent and Influenced by Large Inter-Individual Differences in Younger Adults**

Elly Francis-Pester^1^, Jessica E Manousakis^1^, Anna W.T. Cai^1^, Jinny Collet^1^ & Clare Anderson^1,2^

^1^ School of Psychological Sciences, Monash University, Clayton, VIC Australia *

^2^ Centre for Human Brain Health, School of Psychology, University of Birmingham, Edgbaston, UK

*Work was performed here

**Running Head:** Sleep deprivation, ageing and performance impairment

**Corresponding Author:**

Clare Anderson, PhD.

Centre for Human Brain Health,

School of Psychology,

University of Birmingham,

UK

Email: c.anderson.4@bham.ac.uk

**Supplementary Data**

**S1. Effect of age and sleep deprivation on traditional PVT metrics at the group level.**

*Mean RT and Number of Lapses*

Comparable findings were observed for Mean RT of all responses that we describe for mean RT of timely responses, that is a main effect of TSW (*F* _5,63.21_=21.34, *p* < 0.001), no main effect of age (*p* = 0.980), and no age x TSW interaction (*p* = 0.228). Comparable findings for standard PVT lapse metrics (all lapses > 500ms) were also found: Main effect of TSW (*F* _5,65_=27.0, *p* < 0.001), yet no main effect of age (*p* = 0.667) or age x TSW interaction (*p* = 0.611).

*PVT Variability: Standard Deviation*

PVT Variability reflects wake-state instability and has previously shown age-related differences. Given it reflects both sleep initiation and non-sleep initiation metrics (e.g., fastest versus slowest of responses), we examined it separately to other measures. No interaction between age and TSW was observed (*p* = 0.281), although there was a main effect of TSW (*F*_5,72.57_=12.37, *p* < 0.001) and age (*F*_1,35.35=8.02_, *p* = 0.008), such that younger adults exhibited greater variability overall than older adults.

**S2. Effect of age and sleep deprivation on traditional PVT metrics at the individual level – influence of sex.**

**Figure.** **Individual differences in the response to sleep loss for younger and older adults for common PVT metrics.** [First row] Data represents the mean change score for the average of the well-rested hours (≤16 hours awake) compared to the average of sleep-deprived hours (≥17 hours) for common metrics presented in prior ageing-sleep deprivation literature. All outcomes include individual datapoints, with group mean (dotted line) and 1SD (+/- as grey shading). Darker coloured lines represent females and lighter lines represent males in either age group. [Second row] Mean change by age and sex following sleep deprivation. Data represents the change score from the well-rested hours (≤16 hours awake) compared to the sleep-deprived hours (≥17 hours). All outcomes are presented as Mean (SEM), with effect sizes (Hedges *g*).
